# Understanding the influence of temporary neurologic dysfunction in the outcomes of aortic surgery

**DOI:** 10.1016/j.xjon.2025.09.031

**Published:** 2025-10-06

**Authors:** Valeria Jimenez, Kavya Rajesh, Connor Barrett, Megan Chung, Yanling Zhao, Paul Kurlansky, Joshua Willey, Adham Elmously, Thomas O'Donnell, Virendra Patel, Hiroo Takayama

**Affiliations:** a Division of Cardiothoracic and Vascular Surgery, New York Presbyterian Hospital, Columbia University Medical Center, New York, NY; b Division of Neurology, New York Presbyterian Hospital, Columbia University Medical Center, New York, NY

**Keywords:** neurologic dysfunction, delirium, TIA, stroke, postoperative complications, aortic surgery

## Abstract

**Objective:**

Temporary neurologic dysfunction (ND) is common after aortic surgery. The Valve Academic Research Consortium 3 classifies ND as NeuroARC Type 1 (stroke), Type 2 (covert injury), and Type 3 (transient ischemic attack/delirium without injury). This study applies these definitions to aortic surgery, focusing on Type 3.

**Methods:**

A single-center retrospective analysis of adult patients with open thoracic aortic surgery from March 2005 to December 2023 was performed. Primary end points were mortality and major postoperative complications (reoperation for bleeding, respiratory failure, and acute renal failure). Propensity score weighting using overlap weights balanced covariates between Type 3 and no ND groups. Kaplan-Meier curves and Cox regression analyzed mortality. Multivariable logistic regression identified factors associated with Type 3 ND.

**Results:**

Of 2432 patients, 103 (4.2%) had Type 1, 216 (8.9%) Type 3, and 2113 (86.9%) had no ND. Median age was 62 years (range, 52-71 years), 609 (25.0%) were women, and 1839 (75.6%) underwent aneurysm repair. After balancing, major postoperative complication rates were 49.2% versus 27.6% in Type 3 and no ND, respectively (*P* < .001). There was no difference in 11-year survival (*P* = .943) and Type 3 was not independently associated with mortality. Variables associated with Type 3: age (OR 1.05, 1.048; *P* < .001), left ventricular ejection fraction (OR 0.98, 0.984; *P* = .018), cerebrovascular disease (OR 2.01, 2.011; *P* = .001), cardiopulmonary bypass time in minutes (OR 1.004, 1.004; *P* = .002), retrograde cerebral perfusion (OR 4.25, 4.251; *P* < .001), and major postoperative complications (OR 3.67, *P* < .001).

**Conclusions:**

Type 3 occurs in about 10% of cases and is associated with in-hospital complications but not mortality. Identified risk factors may aid in prevention.

## Graphical Abstract

**Figure:**
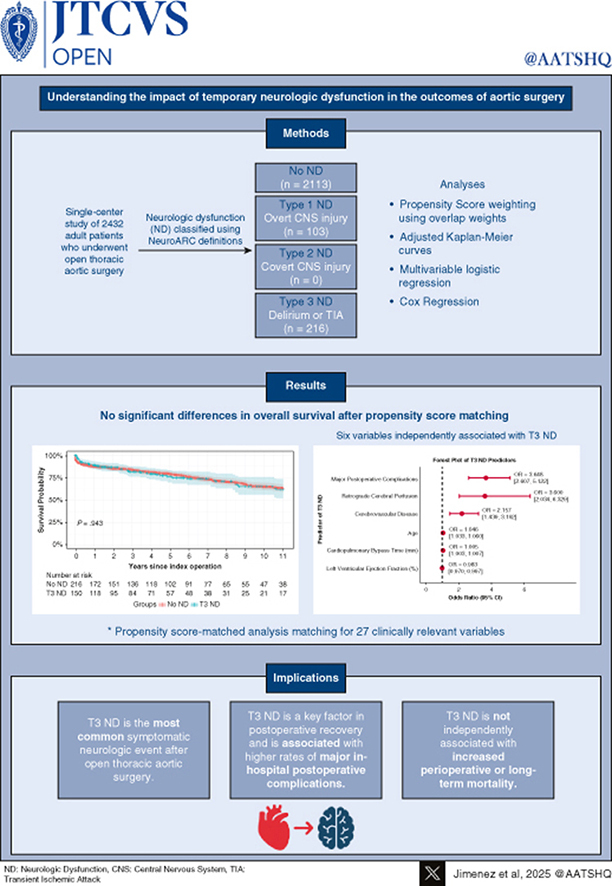

**Figure:**
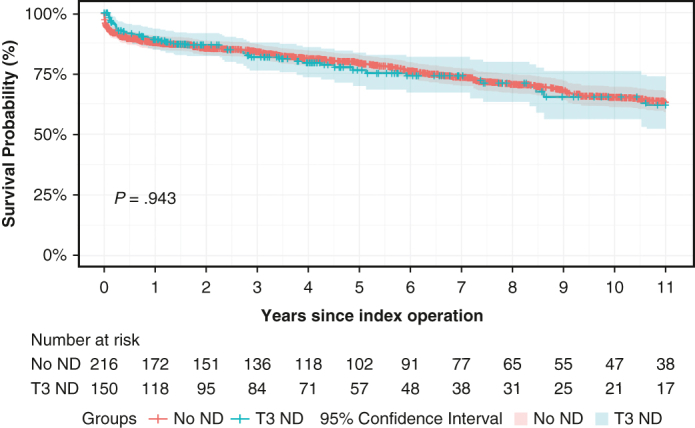
Adjusted long-term survival curve. 95% CI.

**Central Message**

Patients undergoing aortic surgery commonly develop T3 neurologic dysfunction, which is associated with postoperative complications but not mortality.

**Perspective**

T3 neurologic dysfunction, including TIA and delirium, is the most common symptomatic neurologic event after aortic surgery. It is not associated with mortality but is linked to postoperative complications and may influence recovery. Applying standardized criteria such as NeuroARC may improve recognition, risk stratification, and management.

Postoperative neurologic dysfunction (ND) is a serious complication following open thoracic aortic surgery. Postoperative stroke occurs in up to 13% of cases and is associated with long-term disability, in-hospital mortality, and reduced long-term survival.^1^^,^^2^

However, ND goes beyond stroke and includes transient ischemic attack (TIA) and postoperative delirium. Postoperative TIA and postoperative delirium occur in 2.6% and 34% of aortic surgery patients, respectively.^2^^,^^3^ Although associated with cognitive decline, prolonged hospitalization, readmission, and mortality, TIA and postoperative delirium literature remain limited.^4^, ^5^, ^6^

Recognizing the need for a standardized framework, the Valve Academic Research Consortium-3 (VARC-3) introduced an updated neurologic end point classification with 3 NeuroARC categories: Type 1 (T1), overt strokes confirmed by imaging; Type 2 (T2), covert central nervous system injuries detected on imaging without symptoms; and Type 3 (T3), ND without central nervous system injury, including TIA (Type 3a) and delirium (Type 3b).^7^ A prior study applying NeuroARC criteria after transcatheter aortic valve replacement found that 16.1% of patients experienced neurologic events, 58.0% of which were T3.^8^ This study aims to apply the VARC-3 criteria to classify T3 and assess preoperative risk factors, postoperative complications, and patient outcomes following aortic surgery.

## Materials and Methods

This retrospective study was approved by the Columbia University Medical Center Institutional Review Board (AAAR2949; approved November 20, 2024). Given the retrospective design, individual patient consent was waived.

### Patient Selection

**Figure 1:**
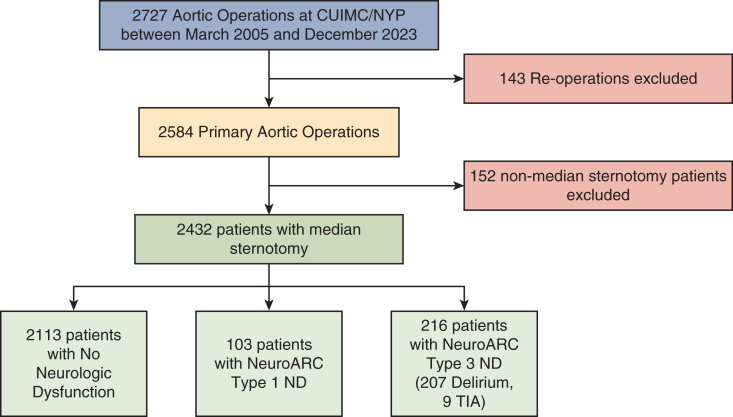
Consolidated Standards of Reporting Trails diagram.

We retrospectively reviewed the records of patients who underwent thoracic aortic operations between March 2005 and December 2023 at New York-Presbyterian Hospital/Columbia University Irving Medical Center. Exclusion criteria included reoperation (n = 143), to reduce potential confounding from altered anatomy and increased surgical complexity, and nonmedian sternotomy procedures (n = 152). Among the 2432 patients remaining, 13.1% (n = 319) had postoperative ND, including 4.2% (n = 103) T1, 0 T2 (reflecting our institutional practice of brain imaging only when clinically indicated), and 8.9% (n = 216) T3 (Figure 1). Delirium was defined using Diagnostic and Statistical Manual of Mental Disorders 5 delirium criteria.^9^

### Data Collection and Definitions

Baseline demographics, patient characteristics, procedural details, postoperative outcomes, and ND details were collected from the Columbia University Irving Medical Center Aortic Center Database and electronic medical records. Missing data were imputed using the random forest algorithm (Table E1). All-cause mortality during the follow-up period was collected through clinical encounters and supplemented through the Centers for Disease Control and Prevention National Death Index, ensuring a 100% follow-up effort on alive/dead status through December 31, 2022.^10^ Variables were defined per Society of Thoracic Surgeons Adult Cardiac Database when applicable.^11^ ND was defined as any new-onset neurologic postoperative event during index hospitalization and was categorized per NeuroARC. Classification was determined through chart review, incorporating neurologic assessments and consults when available. Although not consistently adjudicated by neurology, classification followed VARC-3, which permits nonneurologist assessment when imaging or formal evaluation is unavailable.

### Surgical Procedures and Patient Management

The indication for surgery was determined by the attending surgeon based on established guidelines.^12^ For patients undergoing aortic root replacement, the decision to spare the native valve was based on the integrity of the aortic valve and existing comorbidities.^13^ For root and valve replacement, the choice of mechanical or biological prosthesis or Ross procedure also considered patient preference. For aortic arch replacement, the approach to cerebral protection was determined by the operating surgeon. Antegrade cerebral perfusion (ACP) was performed under moderate hypothermia (28 °C) via axillary or direct innominate artery cannulation and retrograde cerebral perfusion (RCP) under deep hypothermia (20 °C) through the superior vena cava cannula.^14^

Postoperatively, patients were continuously monitored in the intensive care unit and step-down unit. Neurological status was assessed hourly in the intensive care unit and every 8 hours in the step-down unit. Any new-onset ND was evaluated by the unit and cardiac surgery teams. Neurology and/or psychiatry was consulted per the primary team's discretion. Patients with neurologic signs were evaluated per institutional “stroke code,” including urgent computed tomography and intervention when indicated. Magnetic resonance imaging was performed selectively based on clinical judgment, consistent with institutional and literature-based protocols discouraging routine imaging unless focal deficits or signs of acute central nervous system pathology were present.^15^ Delirium was managed with hemodynamic support, reduced sedation, safety measures, and routine assessments. Patients with persistent deficits received comprehensive evaluations before discharge and received outpatient management.

### Statistical Analysis

**Figure E1:**
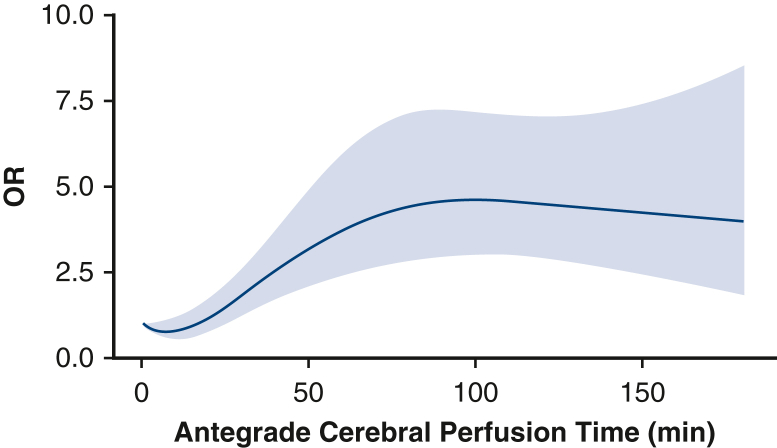
Cubic spline analysis for antegrade cerebral perfusion time.

**Figure E2:**
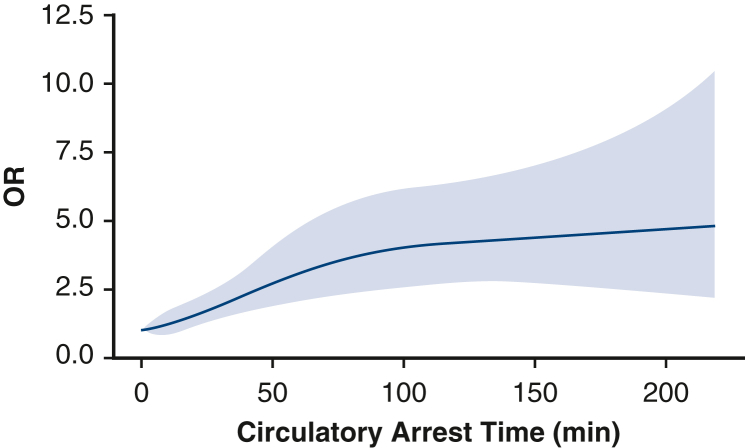
Cubic spline analysis for circulatory arrest time.

Categorical variables were reported as frequencies with percentages and compared using χ^2^ or Fisher exact test. Continuous variables were assessed as nonnormally distributed by the Shapiro-Wilk test, reported as medians with interquartile ranges, and compared using the Mann-Whitney *U* test. Cutoffs for continuous variables were clinically defined and evaluated with cubic splines (Figures E1 and E2). Multivariable logistic regression identified factors associated with major postoperative complications (reoperation for bleeding, respiratory failure, and acute renal failure) and perioperative mortality (in-hospital or within 30 days). Variables that were not ideally balanced after matching were included in the regression models to account for the potential of residual confounding. Kaplan-Meier analysis with log-rank test compared survival between groups. Cox regression assessed factors associated with 11-year mortality and met proportional hazards assumptions. Variables for all models were selected based on clinical judgment and univariable analyses. Stepwise regression was used for logistic models. Collinearity was assessed via variance inflation factor (<5 acceptable).

Patients without ND were compared with T3 using propensity-score overlap weighting analyses. Propensity scores were generated for T3 versus no ND based on the logistic regression model. Variables included in the model: age, sex, body mass index, hypertension, dyslipidemia, diabetes mellitus (DM), dialysis, chronic obstructive pulmonary disease, coronary artery disease, peripheral artery disease, prior myocardial infarction, congestive heart failure, preoperative atrial fibrillation, cerebrovascular disease (CVD), prior coronary intervention, left ventricular ejection fraction (LVEF), aortic aneurysm, distal extension/arch procedure, concomitant mitral/tricuspid valve procedure or coronary artery bypass graft, cardiopulmonary bypass (CPB) time, crossclamp time, aortic cannulation site, lowest body temperature >32 °C, cerebral perfusion, ACP time >80 minutes, lower body circulatory arrest time >100 minutes, and deep hypothermic circulatory arrest. Intraoperative factors were included in the matching algorithm given that they preceded the occurrence of postoperative ND. Overlap weights assigned patients the inverse group probability, ensuring propensity score overlap and covariate balance. Balance in the matching algorithm was determined by a standard mean difference <0.1. A threshold alpha level of 0.05 was used to determine statistical significance. R statistical software version 4.4.0 (R Foundation for Statistical Computing) was used for all the analysis and figures.

## Results

### Patient Baseline Characteristics and Operative Details

For the comparison of no ND versus T3 groups, 103 patients with T1 were excluded. Of 2329 patients remaining, 216 (9.27%) developed T3. Baseline characteristics are presented in Table 1. Compared with those without ND, T3 patients were older (69.0 vs 61.0 years; *P* < .001) and had higher rates of comorbidities, including hypertension, DM, peripheral artery disease, vascular disease, atrial fibrillation, and CVD. T3 patients more frequently underwent emergency (22.7% vs 7.0%; *P* < .001) and urgent procedures (30.6% vs 20.9%; *P* < .001), with higher incidence of dissection (28.7% vs 11.3%; *P* < .001).

**Table 1:** Patient demographics

Variable	Prebalancing			Postbalancing			
	No ND (n = 2113)	Type 3 ND (n = 216)	*P* value	No ND (n = 216.1)	Type 3 ND (n = 150.1)	*P* value	SMD
Age (y)	61.00 (51.00-70.00)	69.00 (61.00-77.00)	<.001	69.00 (60.00-76.00)	68.38 (59.95-76.91)	.919	0.048
Female sex (%)	525 (24.8)	53 (24.5)	.986	55.5 (25.7)	39.8 (26.5)	.815	0.019
Body mass index	27.18 (24.49-30.88)	27.95 (24.91-31.80)	.062	27.69 (25.06-31.68)	27.85 (24.74-31.33)	1.00	0.004
Hypertension (%)	1479 (70.0)	178 (82.4)	<.001	176.0 (81.4)	120.5 (80.3)	.705	0.03
Dyslipidemia (%)	1129 (53.4)	119 (55.1)	.693	118.5 (54.8)	82.0 (54.6)	.951	0.005
Diabetes mellitus (%)	251 (11.9)	50 (23.1)	<.001	45.4 (21.0)	28.7 (19.1)	.542	0.048
Dialysis (%)	27 (1.3)	6 (2.8)	.14	5.8 (2.7)	3.9 (2.6)	.924	0.008
Creatinine (mg/dL)	0.99 (0.83-1.14)	1.09 (0.89-1.36)	<.001	1.00 (0.89-1.21)	1.06 (0.89-1.33)	.039	0.158
COPD (%)	189 (8.9)	23 (10.6)	.481	23.5 (10.9)	16.5 (11.0)	.969	0.003
Coronary artery disease (%)	990 (46.9)	113 (52.3)	.144	115.5 (53.4)	81.1 (54.0)	.884	0.011
Peripheral artery disease (%)	274 (13.0)	45 (20.8)	.002	42.8 (19.8)	28.2 (18.8)	.74	0.026
Congestive heart failure (%)	1179 (55.8)	134 (62.0)	.091	133.7 (61.9)	92.3 (61.5)	.925	0.007
Vascular desease (%)	352 (16.7)	69 (31.9)	<.001	61.8 (28.6)	40.6 (27.0)	.658	0.035
Preoperative atrial fibrillation (%)	408 (19.3)	58 (26.9)	.011	56.1 (26.0)	37.6 (25.1)	.791	0.021
Previous cardiac intervention (%)	540 (25.6)	84 (38.9)	<.001	78.1 (36.1)	50.6 (33.7)	.516	0.051
LVEF	55.00 (53.00-60.00)	55.00 (47.75-58.00)	.005	55.00 (47.06-58.00)	55.00 (48.14-58.00)	.827	0.019
Previous myocardial infarction (%)	96 (4.5)	29 (13.4)	<.001	25.4 (11.8)	15.4 (10.2)	.544	0.049
Cerebrovascular disease (%)	185 (8.8)	47 (21.8)	<.001	40.9 (18.9)	24.6 (16.4)	.402	0.066
Preoperative CVA (%)	131 (6.2)	36 (16.7)	<.001	29.0 (13.4)	18.5 (12.3)	.684	0.032
Operative status (%)			<.001			.023	0.214
Elective	1524 (72.1)	101 (46.8)		128.8 (59.6)	77.0 (51.3)		
Emergency	148 (7.0)	49 (22.7)		28.1 (13.0)	30.6 (20.4)		
Urgent	441 (20.9)	66 (30.6)		59.2 (27.4)	42.6 (28.4)		
Surgical indication (%)			<.001			.12	0.206
Aortic aneurysm	1638 (77.5)	132 (61.1)		137.7 (63.7)	99.4 (66.2)		
Aortic dissection	239 (11.3)	62 (28.7)		49.9 (23.1)	37.3 (24.8)		
Type A (%)			.467			.292	0.184
Acute	195 (9.2)	53 (24.5)		38.9 (18.0)	31.1 (20.7)		
Chronic	43 (2.0)	8 (3.7)		11.0 (5.1)	5.4 (3.6)		
Valvular dysfunction/aortic valve related	166 (7.9)	9 (4.2)		19.9 (9.2)	6.7 (4.4)		
Infection/infective endocarditis	63 (3.0)	12 (5.6)		7.7 (3.5)	6.6 (4.4)		
Other	7 (0.3)	1 (0.5)		1.0 (0.4)	0.1 (0.1)		

Values are presented as median (interquartile range) or n (%). *ND*, Neurologic dysfunction; *SMD*, standardized mean difference; *COPD*, chronic obstructive pulmonary disease; *LVEF*, left ventricular ejection fraction; *CVA*, cerebrovascular accident.

Operative details (Table 2) revealed more aortic arch replacements among T3 patients, including hemiarch (19.4% vs 11.9%; *P* < .001) and partial/total arch (23.1% vs 11.5%; *P* < .001), and more concomitant mitral/tricuspid valve repair (15.7% vs 7.6%; *P* < .001). T3 patients had longer CPB (182.5 vs 142.0 minutes; *P* < .001), crossclamp (119.0 vs 103.0 minutes; *P* < .001), circulatory arrest (26.0 vs 17.0 minutes; *P* < .001), and ACP times (24.0 vs 14.0 minutes; *P* < .001), and lower lowest body temperature (28.25 vs 32.0 °C; *P* < .001).

**Table 2:** Operative characteristics

Variable	Prebalancing			Postbalancing			
	No ND (n = 2113)	Type 3 ND (n = 216)	*P* value	No ND (n = 216.1)	Type 3 ND (n = 150.1)	*P* value	SMD
Isolated ascending aorta replacement (%)	99 (4.7)	9 (4.2)	.861	6.0 (2.8)	7.0 (4.7)	.16	0.101
Aortic valve replacement/repair (%)			.091			.644	0.105
None	960 (45.4)	93 (43.1)		96.1 (44.5)	67.1 (44.7)		
Bioprosthetic valve	638 (30.2)	81 (37.5)		72.0 (33.3)	55.1 (36.7)		
Mechanical valve	64 (3.0)	3 (1.4)		5.0 (2.3)	2.3 (1.5)		
Repair	451 (21.3)	39 (18.1)		43.0 (19.9)	25.7 (17.1)		
Aortic root replacement/repair (%)			.073			.259	0.192
None	354 (16.8)	51 (23.6)		45.1 (20.9)	32.8 (21.9)		
VSRR	451 (21.3)	40 (18.5)		39.0 (18.0)	29.7 (19.8)		
Bioprosthetic Bentall	525 (24.8)	60 (27.8)		52.4 (24.2)	43.8 (29.2)		
Mechanical Bentall	740 (35.0)	63 (29.2)		74.2 (34.4)	42.2 (28.1)		
Ross	6 (0.3)	0 (0.0)		0.3 (0.1)	0.0 (0.0)		
Homograft	37 (1.8)	2 (0.9)		5.1 (2.4)	1.6 (1.1)		
Aortic arch replacement (%)			<.001			.852	0.045
None	1618 (76.6)	124 (57.4)		128.2 (59.3)	92.2 (61.4)		
Hemiarch	252 (11.9)	42 (19.4)		41.5 (19.2)	27.9 (18.6)		
Partial/total arch	243 (11.5)	50 (23.1)		46.4 (21.5)	30.0 (20.0)		
Elephant trunk procedure (%)			.052			.442	0.108
None	2054 (97.2)	204 (94.4)		202.9 (93.9)	141.5 (94.3)		
Conventional elephant trunk	38 (1.8)	9 (4.2)		6.6 (3.0)	6.1 (4.1)		
Frozen elephant trunk	21 (1.0)	3 (1.4)		6.6 (3.0)	2.4 (1.6)		
Concomitant CABG (%)			.497			.712	0.067
None	1475 (69.8)	157 (72.7)		151.0 (69.9)	109.0 (72.6)		
1-2	495 (23.4)	43 (19.9)		46.9 (21.7)	28.6 (19.1)		
3+	143 (6.8)	16 (7.4)		18.1 (8.4)	12.5 (8.3)		
Concomitant mitral or tricuspid valve repair (%)	160 (7.6)	34 (15.7)	<.001	24.0 (11.1)	20.6 (13.7)	.285	0.08
Cardiopulmonary bypass time (min)	142.00 (110.00-184.00)	182.50 (139.00-231.00)	<.001	173.00 (131.00-223.00)	170.25 (132.00-216.22)	.551	0.089
Crossclamp time (min)	103.00 (78.00-136.00)	119.00 (84.75-160.50)	<.001	116.00 (86.00-155.00)	112.96 (81.19-150.00)	.386	0.063
Arterial cannulation site (%)			.017			.56	0.117
Aorta	1406 (66.5)	135 (62.5)		135.7 (62.8)	94.8 (63.2)		
Axillary	605 (28.6)	66 (30.6)		65.8 (30.4)	44.3 (29.5)		
Femoral	41 (1.9)	11 (5.1)		7.1 (3.3)	7.7 (5.1)		
Innominate/brachiocephalic	61 (2.9)	4 (1.9)		7.5 (3.5)	3.3 (2.2)		
Lowest body temperature (°C)	32.00 (28.00-32.00)	28.25 (24.00-32.00)	<.001	28.00 (24.80-32.00)	29.00 (24.00-32.00)	.948	0.004
Circulatory arrest (%)	872 (41.3)	127 (58.8)	<.001	127.8 (59.1)	81.4 (54.2)	.206	0.099
Circulatory arrest time	17.00 (11.00-29.25)	26.00 (15.00-56.65)	<.001	21.00 (13.00-43.00)	24.00 (14.00-39.28)	.489	0.028
Cerebral perfusion (%)	818 (38.7)	126 (58.3)	<.001	120.6 (55.8)	80.0 (53.3)	.52	0.051
Cerebral perfusion type (%)			<.001			.007	0.269
None	1295 (61.3)	90 (41.7)		95.5 (44.2)	70.1 (46.7)		
CP	584 (27.6)	67 (31.0)		76.2 (35.2)	43.0 (28.6)		
ACP time (min)	14.00 (10.00-23.00)	24.00 (17.00-68.50)	<.001	17.00 (12.00-40.00)	21.59 (16.00-43.34)	.003	0.11
RCP	99 (4.7)	35 (16.2)		17.2 (8.0)	23.0 (15.3)		
RCP time (min)	6.00 (4.00-11.00)	8.00 (4.00-15.00)	.103	6.00 (4.00-12.00)	8.00 (4.00-15.00)	.138	0.053
Both	135 (6.4)	24 (11.1)		27.2 (12.6)	14.0 (9.3)		
DHCA (%)	153 (7.2)	38 (17.6)	<.001	36.4 (16.9)	23.9 (15.9)	.757	0.025
DHCA time (min)	12.00 (1.00-23.00)	7.00 (1.00-25.50)	.6	14.16 (1.00-27.19)	11.01 (1.00-33.02)	.679	0.114

Values are presented as median (interquartile range) or n (%). *ND*, Neurologic dysfunction; *SMD*, standardized mean difference; *CABG*, coronary artery bypass graft; *ACP*, antegrade cerebral perfusion; *RCP*, retrograde cerebral perfusion; *DHCA*, deep hyperthermic circulatory arrest.

### Isolated T3 ND: Propensity Score Matching

Appendix E1 highlights unadjusted survival analysis. Propensity score weighting using overlap weights created well-balanced groups of 216 no ND and 150 T3 patients (Table 3). In-hospital outcomes are shown in Table 4. In the matched cohort excluding T1, T3 patients had higher rates of major postoperative complications (57.6% vs 35.5%; *P* < .001), longer ventilation times (median time, 29.25 vs 12.8 hours; *P* < .001), and higher incidence of respiratory (52.0% vs 28.9%; *P* < .001) and renal failure (19.9% vs 14.2%; *P* = .049).

**Table 3:** Variables used in propensity score logistic regression model using overlapping weights

Variable	No ND (n = 216.09)	Type 3 ND (n = 150.12)	*P* value	SMD
Age (y)	69.00 (60.00-76.00)	68.38 (59.95-76.91)	.919	0.048
Female sex (%)	55.5 (25.7)	39.8 (26.5)	.815	0.019
Body mass index (%)			.567	0.084
<25	52.5 (24.3)	40.2 (26.8)		
25-30	90.2 (41.7)	56.7 (37.8)		
>30	73.4 (34.0)	53.2 (35.5)		
Hypertension (%)	176.0 (81.4)	120.5 (80.3)	.705	0.03
Dyslipidemia (%)	118.5 (54.8)	82.0 (54.6)	.951	0.005
Diabetes (%)	45.4 (21.0)	28.7 (19.1)	.542	0.048
Dialysis (%)	5.8 (2.7)	3.9 (2.6)	.924	0.008
COPD (%)	23.5 (10.9)	16.5 (11.0)	.969	0.003
Coronary artery disease (%)	115.5 (53.4)	81.1 (54.0)	.884	0.011
Peripheral artery disease (%)	42.8 (19.8)	28.2 (18.8)	.74	0.026
Previous myocardial infarction (%)	25.4 (11.8)	15.4 (10.2)	.544	0.049
Congestive heart failure (%)	133.7 (61.9)	92.3 (61.5)	.925	0.007
Preoperative atrial fibrillation (%)	56.1 (26.0)	37.6 (25.1)	.791	0.021
Cerebrovascular disease (%)	40.9 (18.9)	24.6 (16.4)	.402	0.066
Previous cardiac intervention (%)	78.1 (36.1)	50.6 (33.7)	.516	0.051
LVEF	55.00 (47.06-58.00)	55.00 (48.14-58.00)	.827	0.019
Aneurysm surgical indication (%)	137.7 (63.7)	99.4 (66.2)	.511	0.052
Distal extension/arch procedure (%)	87.9 (40.7)	57.9 (38.6)	.588	0.043
Concomitant mitral or tricuspid repair or CABG (%)	82.4 (38.1)	56.3 (37.5)	.868	0.013
Cardiopulmonary bypass time	173.00 (131.00-223.00)	170.25 (132.00-216.22)	.551	0.089
Crossclamp time	116.00 (86.00-155.00)	112.96 (81.19-150.00)	.386	0.063
Aortic cannulation site (%)	135.7 (62.8)	94.8 (63.2)	.923	0.008
Lowest body temperature >32 °C (%)	141.5 (65.5)	95.5 (63.6)	.624	0.039
Cerebral perfusion (%)	120.6 (55.8)	80.0 (53.3)	.52	0.051
Antegrade cerebral perfusion time >80 min (%)	15.7 (7.3)	8.5 (5.6)	.377	0.067
Circulatory arrest time >100 min (%)	14.6 (6.8)	7.7 (5.1)	.352	0.07
DHCA (%)	36.4 (16.9)	23.9 (15.9)	.757	0.025

Values are presented as median (interquartile range) or n (%). *ND*, Neurologic dysfunction; *SMD*, standardized mean difference; *COPD*, valve-sparing root repair; *LVEF*, left ventricular ejection fraction; *CABG*, coronary artery bypass graft; *DHCA*, deep hyperthermic circulatory arrest.

**Table 4:** Postoperative outcomes

Variable	Prebalancing			Postbalancing			
	No ND (n = 2113)	Type 3 ND (n = 216)	*P* value	No ND (n = 216.1)	Type 3 ND (n = 150.1)	*P* value	SMD
Perioperative mortality (%)	67 (3.2)	12 (5.6)	.1	17.2 (8.0)	7.0 (4.6)	.108	0.137
Major postoperative complications (%)	457 (21.6)	134 (62.0)	<.001	76.7 (35.5)	86.3 (57.6)	<.001	0.452
Reoperation for bleeding (%)	114 (5.4)	19 (8.8)	.058	18.2 (8.4)	11.2 (7.4)	.643	0.036
Hours of ventilation	8.80 (4.67-16.63)	38.58 (12.22-135.48)	<.001	12.80 (5.50-38.74)	29.25 (10.00-113.46)	<.001	0.271
ECMO/postcardiotomy shock (%)	87 (4.1)	12 (5.6)	.412	12.7 (5.9)	8.8 (5.8)	.983	0.002
Deep sternal wound infection (%)	22 (1.0)	6 (2.8)	.057	3.4 (1.6)	4.2 (2.8)	.312	0.081
Respiratory failure (%)	340 (16.1)	123 (56.9)	<.001	62.6 (28.9)	78.0 (52.0)	<.001	0.483
Renal failure (%)	163 (7.7)	46 (21.3)	<.001	30.6 (14.2)	29.9 (19.9)	.049	0.153
Renal failure requiring dialysis (%)	55 (2.6)	19 (8.8)	<.001	11.1 (5.1)	12.2 (8.2)	.108	0.121
Pacemaker implantation (%)	108 (5.1)	17 (7.9)	.120	14.2 (6.6)	10.0 (6.6)	.986	0.001
Postoperative day of pacemaker implantation	7.00 (5.00-11.00)	17.50 (8.50-24.50)	.001	8.88 (5.89-14.18)	13.84 (7.00-23.99)	.111	0.702

Values are presented as median (interquartile range) or n (%). *ND*, Neurologic dysfunction; *SMD*, standardized mean difference; *ECMO*, extracorporeal membrane oxygenation.

**Figure 2:**
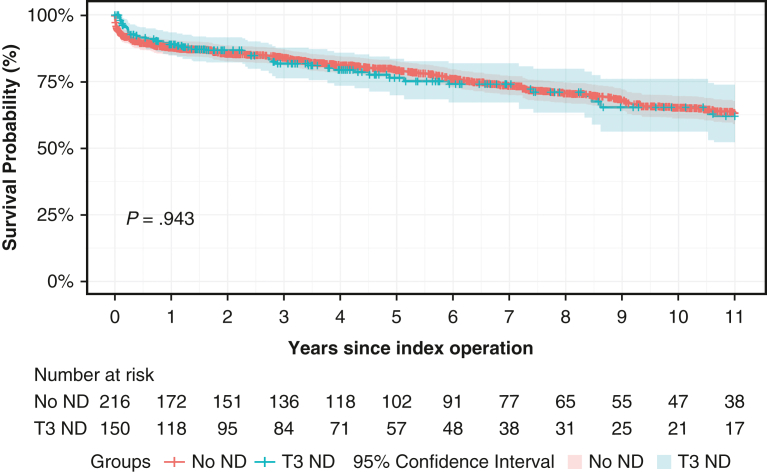
Adjusted long-term survival curve. 95% CI.

T3 was associated with major postoperative complications (odds ratio [OR], 2.468; 95% CI, 1.592-3.828; *P* < .001), but not with perioperative mortality (OR, 0.470; 95% CI, 0.184-1.196; *P* = .113) (Tables E2 and E3). Kaplan-Meier analysis showed no significant difference in adjusted 11-year survival (Figure 2)—T3: 62.1% (95% CI, 52.2%-73.8%) versus no ND: 63.2% (95% CI, 59.0%-67.7%) (*P* = .943). T3 was not associated with decreased 11-year survival (hazard ratio, 0.804; 95% CI, 0.569-1.137; *P* = .217) (Table E4).

### Factors Associated With Postoperative T3 ND

Multivariable logistic regression analysis using the unmatched cohort showed factors independently associated with T3 included age (OR, 1.048; 95% CI, 1.034-1.063; *P* < .001), LVEF (OR, 0.984, 95% CI, 0.97-0.997; *P* = .018), CVD (OR, 2.011; 95% CI, 1.341- 3.042; *P* = .001), CPB time (OR, 1.004; 95% CI, 1.001-1.007; *P* = .002), RCP (OR, 4.251; 95% CI, 2.292-7.671; *P* < .001), and major postoperative complications (OR, 3.667; 95% CI, 2.483-4.988; *P* < .001) (Table E5).

### Characterizing Postoperative T3 ND

Details of postoperative T3 are shown in Tables E6 through E8. Among 216 T3 patients, 207 (95.8%) had delirium and 9 (4.2%) had TIA. The most common delirium symptoms (documented in 189 patients [91.4%]) included agitation/restlessness (55.6%), confusion (40.1%), and hallucinations/delusions (10.6%). Imaging in patients with delirium included head computed tomography in 104 (50.2%) and brain magnetic resonance imaging in 13 (6.3%).

### T1 ND and Aneurysm Repair Subgroup Analysis

Appendix E2 highlights factors independently associated with T1 ND. T1 ND patients had significantly lower 11-year survival than those without ND. Among 103 T1 patients, 33 (32.0%) presented with delirium and 6 had no focal or global deficits but were imaged due to persistent symptoms or other clinical concerns and reclassified based on imaging-confirmed central nervous system injury (Table E9). Appendix E3 provides a subgroup analysis of aneurysm repairs, where T3 ND was associated with higher rates of postoperative complications, but not mortality after adjustment.

## Discussion

Our novel study applies VARC-3 definitions to assess postoperative ND following open thoracic aortic surgery. T3 occurred in 8.9% of patients and was the most common postoperative symptomatic neurologic complication, accounting for 67.7% of all events. T3 was not independently associated with perioperative or long-term mortality but was associated with increased major postoperative complications. Additionally, we reported the clinical details of T3.

T3 is often underrecognized compared with overt events like stroke, yet its prevalence and association with postoperative complications highlight its clinical importance. Although not associated with mortality like T1, T3 was linked to increased morbidity—particularly respiratory failure, prolonged ventilation, and renal failure—which may reflect a bidirectional relationship with ND. ND can delay ventilator weaning, requiring prolonged sedation, whereas extended ventilation may worsen ND through neuroinflammation and neuronal injury.^16^^,^^17^ Similarly, ND may contribute to renal injury via hemodynamic instability, sympathetic activation, and systemic inflammation, whereas renal dysfunction can exacerbate ND by impairing cerebral perfusion.^18^ These findings underscore T3 as a significant perioperative event that may influence recovery and suggest it is more a marker of illness than an independent driver of mortality.

T3 was associated with older age, DM, reduced LVEF, CVD, prolonged CPB time, RCP, and major complications, suggesting intraoperative management plays a role. These associations were identified using multivariable regression in the unmatched cohort to better reflect real-world risk and highlight potentially modifiable contributors. Although T3 ND was not associated with mortality in the matched analysis, worse survival in the unmatched cohort reinforces its clinical significance, supports prevention efforts, and highlights the importance of preoperative and operative risk.

Among patients with T3 ND, postoperative delirium was the most common manifestation, occurring in 64.9% of cases. Hyperactive delirium with agitation, restlessness, and heightened motor activity was the most frequent subtype, followed by hypoactive delirium, characterized by lethargy, inattention, and slowed responses. Mixed delirium presented with fluctuating features of both. Most cases were identified in the intensive care unit rather than the step-down unit, likely reflecting both critical illness and more frequent neurologic monitoring. Symptoms ranged from confusion and hallucinations to lethargy and paranoia, highlighting the need for standardized assessment. TIA was a less common but important manifestation of T3, presenting with transient focal deficits without imaging-confirmed infarction. Imaging in T3 was variable, with head computed tomography as the most common. Neurology and psychiatry consults were also variably obtained, highlighting a gap in multidisciplinary care.

### Limitations

As a retrospective, single-center study conducted at a high-volume aortic center, the generalizability of our findings may be limited. Although this is among the first and largest studies applying VARC-3 ND classification to aortic surgery, it may still be underpowered to detect subtle differences in mortality. Additionally, we could not match for more granular data points, including cerebral malperfusion from acute type A dissection. Follow-up was also limited for key outcomes, including cognitive and functional status, quality of life, and neuropsychiatric outcomes.

Although our clinical approach aligns with current guidelines, which do not recommend neuroimaging for silent cerebrovascular disease outside of research or high-risk populations, or in cases of delirium without focal neurologic deficits or other concerning signs, given that stroke is rarely the etiology of delirium in such cases, the importance of covert central nervous system injury (T2 ND) remains debated.^7^^,^^15^^,^^19^ The absence of T2 ND data in our study reflects recognized challenges in detecting covert central nervous system injuries, including the lack of standardized definitions and inconsistent correlation between magnetic resonance imaging-detected lesions and neurocognitive outcomes, as shown in prior surgical aortic valve replacement and transcatheter aortic valve replacement studies.^7^ However, T2 ND events are increasingly recognized in the literature, and their potential association with long-term cognitive outcomes underscores their clinical relevance and warrants further study despite limitations in routine detection.^20^^,^^21^

This lack of routine imaging may have resulted in some T3 ND cases representing undetected central nervous system injury. However, most patients presenting with delirium who were ultimately determined to have T1 ND also had focal or global deficits warranting imaging to confirm overt central nervous system injury. Additionally, better outcomes in T3 ND compared with adjudicated T1 ND support a clinical distinction. Given that delirium is often variably defined and diagnosed subjectively, we applied Diagnostic and Statistical Manual of Mental Disorders 5 criteria to ensure standardized and validated classification. Although T3 was strongly associated with postoperative complications, the temporal and causal relationship remains unclear. Future prospective studies are needed to clarify these relationships and assess the long-term influence of T3.

## Conclusions

**Figure 3:**
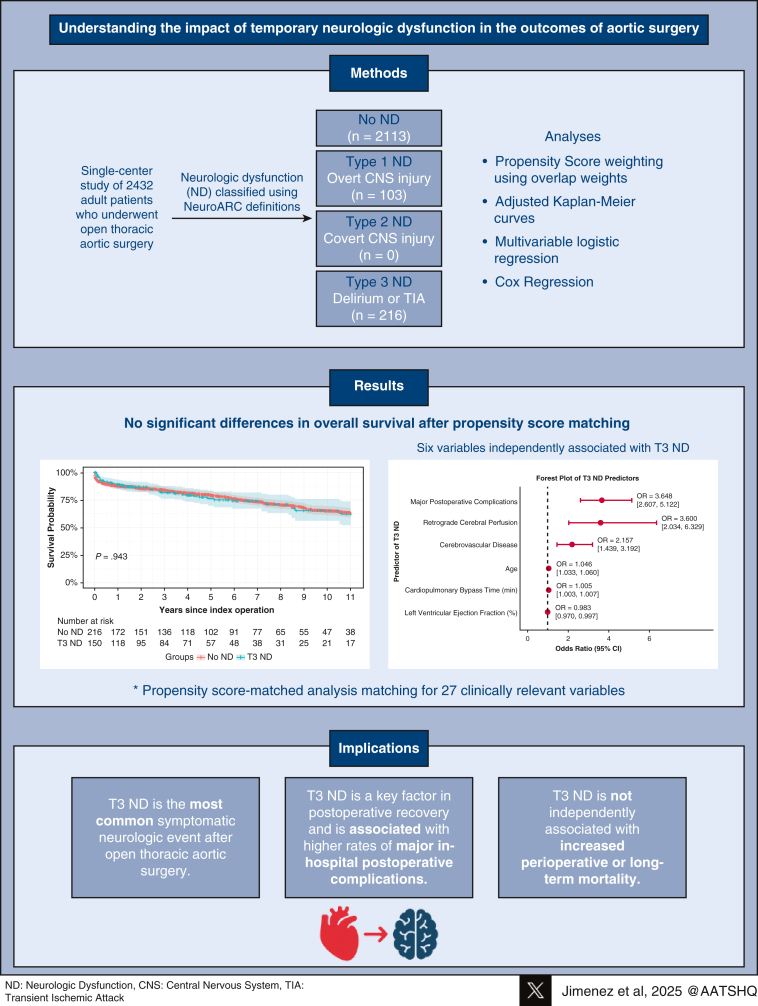
Type 3 neurologic dysfunction after aortic surgery is associated with postoperative complications and comparable short- and long-term survival.

This novel study applies the VARC-3 classification system to evaluate postoperative ND following open thoracic aortic surgery. T3 occurred in 8.9% of patients and was the most frequent neurologic event. It was associated with increased major postoperative complications but not with mortality. These findings underscore T3's clinical relevance and its potential influence on postoperative recovery. See Figure 3 for a graphical abstract of the study.

## Conflict of Interest Statement

The authors reported no conflicts of interest.

The *Journal* policy requires editors and reviewers to disclose conflicts of interest and to decline handling or reviewing manuscripts for which they may have a conflict of interest. The editors and reviewers of this article have no conflicts of interest.

## Figures and Tables

**Figure E3:**
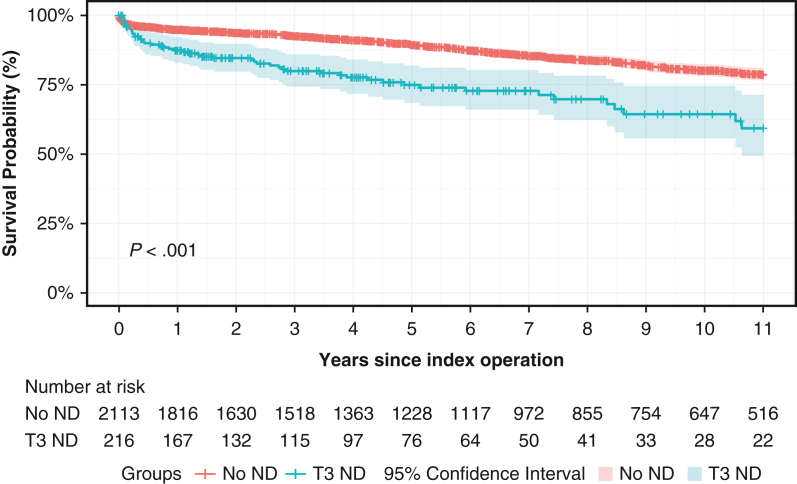
Unadjusted long-term survival curve. 95% Confidence Interval.

**Figure E4:**
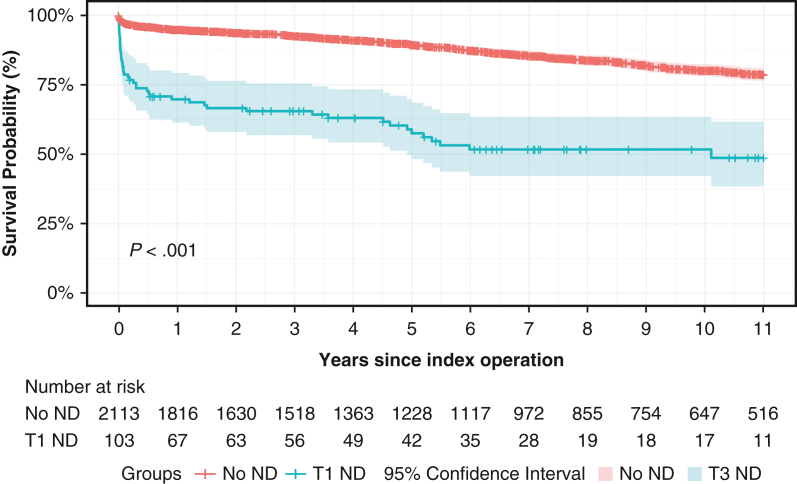
T1 ND long-term survival curve. 95% Confidence Interval.

**Figure E5:**
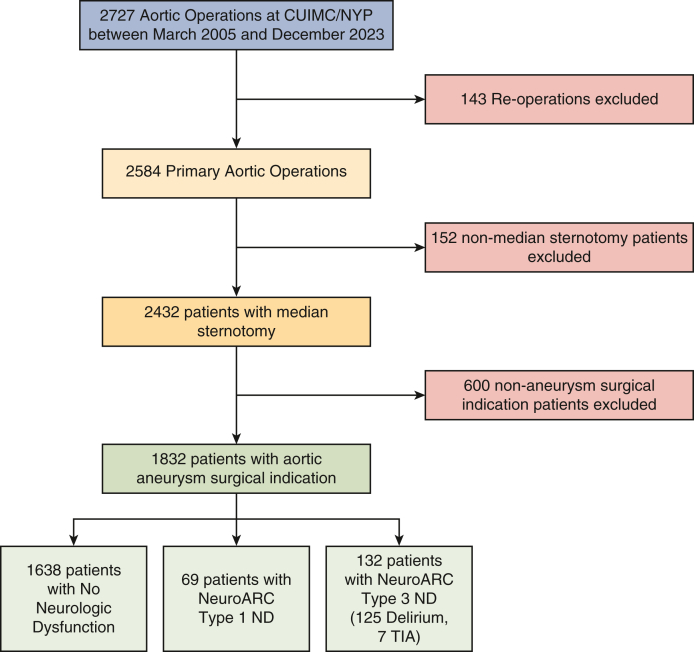
Aneurysm subgroup consort diagram.

**Figure E6:**
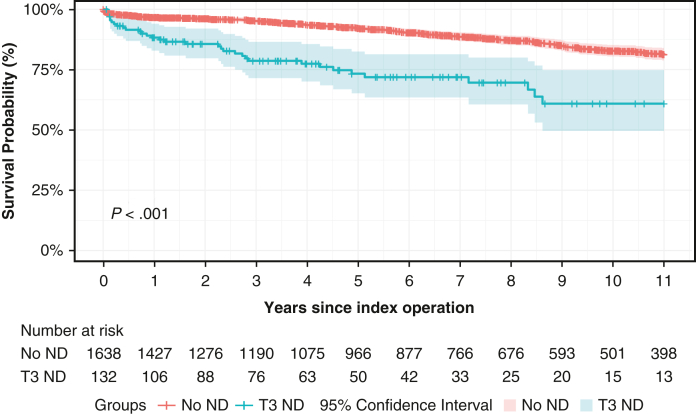
Aneurysm subgroup unadjusted long-term survival curve. 95% CI.

**Figure E7:**
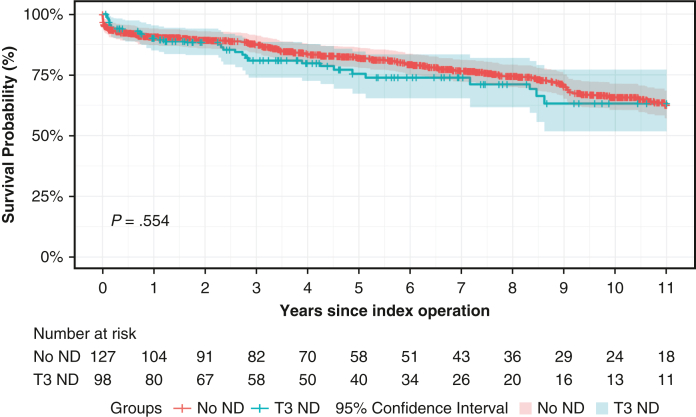
Aneurysm subgroup adjusted long-term survival curve. 95% CI.

**Table E1:** Missing data

Variable	Missingness (%)
Age	0
Sex	0
Hypertension	0.3
Dyslipidemia	0
Diabetes mellitus	0.3
Dialysis	0.3
Vascular disease	0
Bicuspid valve	0
Chronic obstructive pulmonary disease	1.4
Coronary artery disease	0
Peripheral artery disease	0.3
Previous myocardial infarction	0.1
Congestive heart failure	0.5
Pre-operative atrial fibrillation	0.3
Cerebrovascular disease	0.3
Preoperative cerebrovascular accident	0.4
Previous cardiac intervention	0
Operative status	0
Body mass index	0.2
Left ventricular ejection fraction	1.4
Aortic insufficiency	2.1
Aortic stenosis	0.7
Creatinine	1.1
Surgical indication	0.3
Type A dissection	3.2
Cardiopulmonary bypass time	4
Crossclamp time	4.5
Cannulation site	2.4
Lowest body temperature	1.3
Cerebral perfusion	1.4
Antegrade cerebral perfusion time	4.2
Circulatory arrest time	12.2
Circulatory arrest	0.1
Cerebral perfusion type	1.4
Retrograde cerebral perfusion time	3
Deep hypothermic circulatory arrest time	5
Deep hypothermic circulatory arrest	1.9
Thoracic endovascular aortic repair	3
Isolated ascending aortic procedure	1.4
Aortic valve procedure	2.9
Aortic root procedure	0
Aortic arch procedure	2.4
Elephant trunk procedure	0.1
Concomitant coronary artery bypass graft	0
Concomitant mitral/tricuspid valve repair	3.3
Delirium classification	8.3
Delirium presentation location	10.2
Delirium symptoms	7.9
Head computed tomography	0
Brain magnetic resonance imaging	0
Electroencephalogram	0
Neurology consultation	0
Psychiatry consultation	0
Diabetes mellitus	0.3
Dialysis	0.3
Vascular disease	0
Bicuspid valve	0

**Table E2:** Multivariable logistic regression for factors associated with major postoperative complications

Variable	Multivariable analysis		
	Odds ratio	95% CI	*P* value
Type 3 neurologic dysfunction	2.468	1.592-3.828	<.001
Elective operative status	Ref	Ref	Ref
Emergency operative status	1.510	1.177-1.938	<.001
Urgent operative status	1.212	0.732-2.007	.001
No cerebral perfusion	Ref	Ref	Ref
Antegrade cerebral perfusion	1.078	0.521-2.23	.456
Retrograde cerebral perfusion	1.103	0.536-2.271	.840
Antegrade + retrograde cerebral perfusion	1.003	0.986-1.02	.790
Deep hypothermic circulatory arrest time (min)	2.468	1.592-3.828	.738

*Ref*, Reference category.

**Table E3:** Multivariable logistic regression for factors associated with perioperative mortality

Variable	Multivariable analysis		
	Odds ratio	95% CI	*P* value
Type 3 neurologic dysfunction	0.470	0.184-1.196	.113
Elective operative status	Ref	Ref	Ref
Emergency operative status	3.742	1.191-11.75	.024
Urgent operative status	2.338	0.858-6.37	.097
No cerebral perfusion	Ref	Ref	Ref
Antegrade cerebral perfusion	0.843	0.3-2.369	.746
Retrograde cerebral perfusion	1.466	0.423-5.079	.546
Antegrade + retrograde cerebral perfusion	0.328	0.047-2.318	.264
Deep hypothermic circulatory arrest time (min)	1.009	0.986-1.032	.456

*Ref*, Reference category.

**Table E4:** Multivariable Cox regression for factors associated with 11-year mortality

Variable	Cox regression		
	Hazard ratio	95% CI	*P* value
Type 3 neurologic dysfunction	0.804	0.569-1.137	.217
Elective operative status	Ref	Ref	Ref
Emergency operative status	1.267	1.149-1.397	<.001
Urgent operative status	1.109	0.719-1.71	.641
Deep hypothermic circulatory arrest time (min)	1.391	0.974-1.986	.070
Major postoperative complications	2.583	1.899-3.512	<.001

**Table E5:** Multivariable logistic regression analyses for factors associated with NeuroARC Type 3 neurologic dysfunction in the unmatched cohort

Variable	Multivariable analysis		
	Odds ratio	95% CI	*P* value
Age	1.048	1.034-1.063	<.001
Hypertension	0.939	0.629-1.436	.766
Diabetes mellitus	1.440	0.978-2.144	.069
Dialysis	0.762	0.284-2.121	.596
COPD	0.626	0.368-1.031	.076
Coronary artery disease	1.032	0.73-1.446	.855
Peripheral artery disease	1.176	0.785-1.751	.428
Congestive heart failure	1.071	0.772-1.492	.683
Preoperative atrial fibrillation	1.109	0.769-1.585	.576
Previous cardiac intervention	1.125	0.786-1.631	.527
Left ventricular ejection fraction (%)	0.984	0.97-0.997	.018
Cerebrovascular disease	2.011	1.341-3.042	.001
Surgical indication: Aneurysm	Ref	Ref	Ref
Surgical indication: Dissection	1.205	0.779-1.88	.408
Surgical indication: Other	0.778	0.445-1.372	.381
Isolated ascending aorta procedure	Ref	Ref	Ref
Aortic proximal extension procedure	0.793	0.436-1.436	.445
Aortic distal extension procedure	0.896	0.413-1.922	.779
Aortic proximal + distal extension procedure	0.971	0.475, 2.003	.937
Concomitant CABG or mitral or tricuspid valve	0.894	0.641-1.24	.504
Cardiopulmonary bypass time	1.004	1.001-1.007	.002
Aorta cannulation	Ref	Ref	Ref
Axillary cannulation	0.939	0.593-1.438	.782
Femoral cannulation	0.793	0.341-1.863	.593
Innominate/brachiocephalic cannulation	0.550	0.177-1.696	.300
No cerebral perfusion	Ref	Ref	Ref
Antegrade cerebral perfusion	0.952	0.549-1.666	.862
Retrograde cerebral perfusion	4.251	2.292-7.671	<.001
Antegrade + retrograde cerebral perfusion	1.232	0.625-2.496	.555
Lowest body temperature (°C)	1.007	0.956-1.057	.787
Circulatory arrest time (min)	0.997	0.992-1.003	.315
Deep hypothermic circulatory arrest	1.227	0.71-2.114	.464
Major postoperative complications	3.667	2.483-4.988	<.001

*COPD*, Chronic obstructive pulmonary disease; *Ref*, Reference category; *CABG*, coronary artery bypass graft.

**Table E6:** NeuroARC Type 3 neurologic dysfunction delirium subtype characteristics (n = 207)

Variable	Result
Delirium etiology	
Metabolic encephalopathy	8 (3.9)
Substance withdrawal	9 (4.3)
Preoperative stroke	5 (2.4)
Other	2 (1.0)
Uncertain	183 (88.4)
Delirium classification	
Hypoactive	41 (19.8)
Hyperactive	122 (58.9)
Mixed	35 (16.9)
Not documented	9 (4.3)
Delirium presentation location	
ICU	173 (83.6)
SDU	21 (10.1)
Not documented	13 (6.3)
Delirium symptoms	
Agitation/restlessness	115 (55.6)
Confusion	83 (40.1)
Combative	9 (4.3)
Hallucinations/delusions	22 (10.6)
Impulsivity	5 (2.4)
Lethargic	36 (17.4)
Insomnia	13 (6.3)
Paranoia	14 (6.8)
Not documented	18 (8.6)
Head CT	104 (50.2)
1	73 (35.3)
2	17 (8.2)
≥3	14 (6.8)
Brain MRI	13 (6.3)
1	9 (4.3)
2	3 (1.4)
≥3	1 (0.5)
EEG	40 (19.3)
1	4 (1.9)
2	16 (7.7)
≥3	20 (9.7)
Neurology consultation	79 (38.2)
Psychiatry consultation	63 (30.4)

Values are presented as n (%). *ICU*, Intensive care unit; *SDU*, step-down unit; *CT*, computed tomography; *MRI*, magnetic resonance imaging; *EEG*, electroencephalogram.

**Table E7:** NeuroARC Type 3 neurologic dysfunction transient ischemic attack subtype characteristics (n = 9)

Variable	Result
Head CT	5 (55.6)
1	2 (22.2)
2	2 (22.2)
≥3	1 (11.1)
MRI	1 (11.1)
EEG	1 (11.1)
Neurology consultation	4 (44.4)
Psychiatry consultation	1 (11.1)

Values are presented as n (%). *CT*, Computed tomography; *MRI*, magnetic resonance imaging; *EEG*, electroencephalogram.

**Table E8:** NeuroARC Type 3 neurologic dysfunction characteristics (n = 216)

Variable	Result
Head CT	109 (50.5)
1	75 (34.7)
2	19 (8.8)
≥3	15 (9.6)
MRI	14 (6.5)
EEG	41 (19.0)
Neurology consultation	83 (38.4)
Psychiatry consultation	64 (30.0)

Values are presented as n (%). *CT*, Computed tomography; *MRI*, magnetic resonance imaging; *EEG*, electroencephalogram.

**Table E9:** NeuroARC Type 1 neurologic dysfunction characteristics (n = 103)

Variable	Result
Delirium	33 (32.0)
With focal neurologic deficits	25 (24.3)
With global neurologic deficits	2 (1.0)

Values are presented as n (%).

**Table E10:** Multivariable logistic regression analyses for factors associated with NeuroARC Type 1 neurologic dysfunction

Variable	Multivariable analysis		
	Odds ratio	95% CI	*P* value
Age	1.022	1.005-1.04	.013
Hypertension	1.133	0.644-1.994	.664
Diabetes mellitus	1.908	1.139-3.197	.014
Congestive heart failure	1.701	1.044-2.773	.033
Vascular disease	1.693	1.068-2.685	.025
Preoperative atrial fibrillation	1.076	0.654-1.77	.774
Previous cardiac intervention	0.957	0.581-1.575	.861
Cerebrovascular disease	1.736	0.98-3.073	.058
Surgical indication: Aneurysm	Ref	Ref	Ref
Surgical indication: Dissection	0.989	0.57-1.717	.970
Surgical indication: Other	0.661	0.279-1.562	.345
Isolated ascending aorta procedure	Ref	Ref	Ref
Aortic proximal extension procedure	3.220	0.747-13.888	.117
Aortic distal extension procedure	9.751	2.036-46.709	.004
Aortic proximal + distal extension procedure	5.998	1.257-28.62	.025
Concomitant CABG or mitral or tricuspid valve	0.564	0.35-0.908	.018
Cardiopulmonary bypass time	1.005	1.001-1.008	.005
Aortic cannulation site	0.563	0.334-0.949	.031
Lowest body temperature (°C)	1.021	0.96-1.086	.507
No cerebral perfusion	Ref	Ref	Ref
Antegrade cerebral perfusion	0.246	0.121-0.502	<.001
Retrograde cerebral perfusion	0.393	0.148-1.043	.061
Antegrade + retrograde cerebral perfusion	0.330	0.126-0.865	.024
Circulatory arrest	3.444	1.403-8.454	.007

*Ref*, Reference category; *CABG*, coronary artery bypass grafting.

**Table E11:** Patient baseline characteristics of the aneurysm repair subgroup

Variable	Prebalancing			Postbalancing			
	No ND (n = 1638)	Type 3 ND (n = 132)	*P* value	No ND (n = 127.4)	Type 3 ND (n = 97.96)	*P* value	SMD
Age (y)	61.00 (50.00-70.00)	70.00 (61.75-77.00)	<.001	69.00 (61.00-76.00)	69.00 (61.00-77.00)	.888	0.043
Female sex (%)	359 (21.9)	32 (24.2)	.61	30.9 (24.3)	23.8 (24.3)	.996	0.001
Body mass index	27.22 (24.53, 30.86)	27.77 (24.66-31.14)	.541	27.45 (24.80-31.10)	27.74 (24.45-31.11)	.886	0.001
Hypertension (%)	1140 (69.6)	112 (84.8)	<.001	106.0 (83.2)	80.4 (82.0)	.755	0.031
Dyslipidemia (%)	885 (54.0)	69 (52.3)	.765	66.4 (52.1)	51.1 (52.1)	.995	0.001
Diabetes mellitus (%)	192 (11.7)	24 (18.2)	.041	20.8 (16.3)	14.9 (15.2)	.755	0.031
Dialysis (%)	7 (0.4)	0 (0.0)	.975	0.8 (0.7)	0.0 (0.0)	.152	0.115
Creatinine (mg/dL)	0.98 (0.83-1.11)	1.06 (0.89-1.30)	<.001	1.00 (0.89-1.20)	1.05 (0.85-1.28)	.06	0.178
COPD (%)	136 (8.3)	13 (9.8)	.651	12.7 (10.0)	9.8 (10.0)	.991	0.001
Coronary artery disease (%)	842 (51.4)	87 (65.9)	.002	82.9 (65.1)	63.0 (64.3)	.873	0.016
Peripheral artery disease (%)	200 (12.2)	27 (20.5)	.01	24.8 (19.4)	18.3 (18.7)	.856	0.018
Congestive heart failure (%)	914 (55.8)	88 (66.7)	.02	83.9 (65.9)	63.9 (65.3)	.892	0.013
Vascular disease (%)	254 (15.5)	39 (29.5)	<.001	34.5 (27.1)	25.9 (26.4)	.881	0.015
Preoperative atrial fibrillation (%)	308 (18.8)	34 (25.8)	.067	31.8 (25.0)	23.9 (24.4)	.895	0.013
Previous cardiac intervention (%)	342 (20.9)	54 (40.9)	<.001	47.1 (37.0)	33.8 (34.5)	.6	0.052
LVEF	55.00 (53.00, 60.00)	55.00 (49.50-58.00)	.102	55.00 (50.00-58.00)	55.00 (49.32-58.00)	.865	0.003
Previous myocardial infarction (%)	66 (4.0)	15 (11.4)	<.001	13.3 (10.5)	9.6 (9.8)	.835	0.021
Cerebrovascular disease (%)	122 (7.4)	27 (20.5)	<.001	22.8 (17.9)	16.0 (16.3)	.661	0.043
Preoperative CVA (%)	82 (5.0)	19 (14.4)	<.001	15.4 (12.1)	11.7 (11.9)	.951	0.006
Operative status (%)			<.001			.628	0.099
Elective	1335 (81.5)	89 (67.4)		94.6 (74.3)	68.5 (69.9)		
Emergency	10 (0.6)	5 (3.8)		2.7 (2.1)	2.5 (2.6)		
Urgent	293 (17.9)	38 (28.8)		30.1 (23.6)	27.0 (27.5)		

Values are presented as n (%) or median (interquartile range). *ND*, Neurologic dysfunction; *SMD*, standardized mean difference; *COPD*, chronic obstructive pulmonary disease; *LVEF*, left ventricular ejection fraction; *CVA*, cerebrovascular accident.

**Table E12:** Operative details of the study cohorts of the aneurysm repair subgroup

Variable	Prebalancing			Postbalancing			
	No ND (n = 1638)	Type 3 ND (n = 132)	*P* value	No ND (n = 127.4)	Type 3 ND (n = 97.96)	*P* value	SMD
Isolated ascending aorta replacement (%)	91 (5.6)	6 (4.5)	.77	5.1 (4.0)	4.7 (4.8)	.658	0.042
Aortic valve replacement/repair (%)			.078			.957	0.057
None	706 (43.1)	57 (43.2)		52.6 (41.3)	41.9 (42.7)		
Bioprosthetic valve	528 (32.2)	54 (40.9)		50.7 (39.8)	39.4 (40.3)		
Mechanical valve	45 (2.7)	3 (2.3)		2.6 (2.0)	2.1 (2.1)		
Repair	359 (21.9)	18 (13.6)		21.6 (16.9)	14.6 (14.9)		
Aortic root replacement/repair (%)			.095			.284	0.218
None	247 (15.1)	31 (23.5)		24.1 (18.9)	20.1 (20.6)		
VSRR	383 (23.4)	25 (18.9)		24.5 (19.3)	19.7 (20.1)		
Bioprosthetic Bentall	412 (25.2)	36 (27.3)		31.3 (24.6)	28.0 (28.5)		
Mechanical Bentall	573 (35.0)	40 (30.3)		45.4 (35.7)	30.2 (30.8)		
Ross	5 (0.3)	0 (0.0)		0.2 (0.2)	0.0 (0.0)		
Homograft	18 (1.1)	0 (0.0)		1.8 (1.4)	0.0 (0.0)		
Aortic arch replacement (%)			<.001			.73	0.08
None	1329 (81.1)	81 (61.4)		81.0 (63.6)	63.9 (65.2)		
Hemiarch	167 (10.2)	20 (15.2)		22.5 (17.7)	14.5 (14.8)		
Partial/total arch	142 (8.7)	31 (23.5)		23.8 (18.7)	19.6 (20.0)		
Elephant trunk procedure (%)			.003			.693	0.088
None	1598 (97.6)	122 (92.4)		119.0 (93.4)	90.0 (91.9)		
Conventional elephant trunk	29 (1.8)	7 (5.3)		4.7 (3.7)	5.4 (5.5)		
Frozen elephant trunk	11 (0.7)	3 (2.3)		3.7 (2.9)	2.5 (2.6)		
Concomitant CABG (%)			.018			.224	0.173
None	1140 (69.6)	96 (72.7)		90.6 (71.1)	70.6 (72.1)		
1-2	393 (24.0)	21 (15.9)		26.8 (21.1)	15.8 (16.1)		
3+	105 (6.4)	15 (11.4)		9.9 (7.8)	11.5 (11.8)		
Concomitant mitral or tricuspid valve repair (%)	108 (6.6)	19 (14.4)	.002	14.5 (11.4)	12.8 (13.1)	.604	0.05
Cardiopulmonary bypass time (min)	136.00 (106.25-174.00)	169.00 (132.75-221.00)	<.001	163.00 (123.26-211.81)	154.41 (125.88-207.00)	.742	0.071
Crossclamp time (min)	102.00 (77.00-134.00)	116.00 (83.75-151.50)	.005	112.00 (83.00-148.00)	107.91 (80.00-146.34)	.727	0.038
Arterial cannulation site (%)			.621			.731	0.112
Aorta	1154 (70.5)	95 (72.0)		91.5 (71.8)	70.3 (71.7)		
Axillary	411 (25.1)	30 (22.7)		28.2 (22.1)	21.8 (22.2)		
Femoral	18 (1.1)	3 (2.3)		1.7 (1.3)	2.6 (2.6)		
Innominate/brachiocephalic	55 (3.4)	4 (3.0)		6.0 (4.7)	3.3 (3.4)		
Lowest body temperature (°C)	32.00 (28.00-32.10)	31.30 (26.00-32.00)	<.001	30.30 (27.20-32.00)	31.80 (26.86-32.00)	.943	0.014
Circulatory arrest (%)	593 (36.2)	69 (52.3)	<.001	64.7 (50.8)	46.5 (47.4)	.499	0.067
Circulatory arrest time	16.00 (11.00-27.00)	29.00 (14.75-66.00)	<.001	21.00 (13.00-44.26)	27.09 (13.00-48.19)	.376	0.017
Cerebral perfusion (%)	560 (34.2)	67 (50.8)	<.001	61.9 (48.6)	46.2 (47.1)	.765	0.03
Cerebral perfusion type (%)			<.001			.021	0.304
None	1078 (65.8)	65 (49.2)		65.5 (51.4)	51.8 (52.9)		
ACP	408 (24.9)	34 (25.8)		40.5 (31.8)	23.2 (23.7)		
ACP time (min)	13.00 (10.00-19.00)	23.00 (17.00-66.00)	<.001	16.00 (12.00-41.62)	19.89 (15.00-40.32)	.197	0.042
RCP	66 (4.0)	20 (15.2)		8.0 (6.3)	14.1 (14.4)		
RCP time (min)	6.00 (4.00-9.00)	7.00 (4.00-13.00)	.145	6.00 (3.00-9.00)	7.00 (4.00-13.00)	.126	0.057
Both	86 (5.3)	13 (9.8)		13.5 (10.6)	8.8 (9.0)		
DHCA (%)	77 (4.7)	15 (11.4)	.002	13.5 (10.6)	9.9 (10.1)	.868	0.018
DHCA time (min)	10.50 (1.00-18.30)	12.00 (1.50-39.00)	.207	2.00 (1.00-20.58)	11.11 (1.00-38.00)	.248	0.132

Values are presented as n (%) or median (interquartile range). *ND*, Neurologic dysfunction; *SMD*, standardized mean difference; *VSRR*, valve-sparing root repair; *CABG*, coronary artery bypass grafting; *ACP*, antegrade cerebral perfusion; *RCP*, retrograde cerebral perfusion; *DHCA*, deep hypothermic circulatory arrest.

**Table E13:** Propensity score weighting using overlap weights for NeuroARC Type 3 neurologic dysfunction in aneurysm repair subgroups

Variable	No ND (n = 127.85)	Type 3 ND (n = 97.77)	*P* value	SMD
Age (y)	69.00 (61.00-76.00)	69.00 (61.00-77.00)	.888	0.043
Female (%)	30.9 (24.3)	23.8 (24.3)	.996	0.001
Body mass index, kg/m (%)			.75	0.075
<25	34.2 (26.9)	28.3 (28.9)		
25-30	52.0 (40.8)	36.4 (37.1)		
>30	41.2 (32.3)	33.2 (33.9)		
Hypertension (%)	106.0 (83.2)	80.4 (82.0)	.755	0.031
Dyslipidemia (%)	66.4 (52.1)	51.1 (52.1)	.995	0.001
Diabetes (%)	20.8 (16.3)	14.9 (15.2)	.755	0.031
COPD (%)	12.7 (10.0)	9.8 (10.0)	.991	0.001
Coronary artery disease (%)	82.9 (65.1)	63.0 (64.3)	.873	0.016
Peripheral artery disease (%)	24.8 (19.4)	18.3 (18.7)	.856	0.018
Preoperative myocardial infarction (%)	13.3 (10.5)	9.6 (9.8)	.835	0.021
Congestive heart failure (%)	83.9 (65.9)	63.9 (65.3)	.892	0.013
Preoperative atrial fibrillation (%)	31.8 (25.0)	23.9 (24.4)	.895	0.013
Cerebrovascular disease (%)	22.8 (17.9)	16.0 (16.3)	.661	0.043
Preoperative coronary intervention (%)	47.1 (37.0)	33.8 (34.5)	.6	0.052
LVEF	55.00 (50.00-58.00)	55.00 (49.32-58.00)	.865	0.003
Distal extension/arch procedure (%)	46.4 (36.4)	34.1 (34.8)	.736	0.034
Concomitant mitral or tricuspid repair or CABG (%)	47.6 (37.4)	36.3 (37.0)	.946	0.007
Cardiopulmonary bypass time (min)	163.00 (123.26-211.81)	154.41 (125.88-207.00)	.742	0.071
Crossclamp time (min)	112.00 (83.00-148.00)	107.91 (80.00-146.34)	.727	0.038
Aortic cannulation site (%)	91.5 (71.8)	70.3 (71.7)	.982	0.002
Lowest body temperature >32 °C (%)	73.3 (57.6)	55.3 (56.5)	.822	0.022
Cerebral perfusion (%)	61.9 (48.6)	46.2 (47.1)	.765	0.03
Antegrade cerebral perfusion time >80 min (%)	7.5 (5.9)	4.4 (4.4)	.514	0.065
Circulatory arrest time >100 min (%)	6.8 (5.3)	3.9 (4.0)	.539	0.063
Deep hypothermic circulatory arrest (%)	13.5 (10.6)	9.9 (10.1)	.868	0.018

Values are presented as n (%) or median (interquartile range). *ND*, Neurologic dysfunction; *SMD*, standardized mean difference; *COPD*, chronic obstructive pulmonary disease; *LVEF*, left ventricular ejection fraction; *CABG*, coronary artery bypass grafting.

**Table E14:** Postoperative outcomes of the aneurysm repair subgroup

Variable	Prebalancing			Postbalancing			
	No ND (n = 1638)	Type 3 ND (n = 132)	*P* value	No ND (n = 127.4)	Type 3 ND (n = 97.96)	*P* value	SMD
Perioperative mortality (%)	30 (1.8)	7 (5.3)	.018	8.5 (6.7)	4.1 (4.1)	.31	0.112
Major postoperative complications (%)	276 (16.8)	71 (53.8)	<.001	35.1 (27.6)	48.2 (49.2)	<.001	0.457
Reoperation for bleeding (%)	70 (4.3)	10 (7.6)	.124	9.5 (7.5)	5.9 (6.0)	.57	0.058
Hours of ventilation	8.30 (5.00-14.57)	25.20 (10.97-110.08)	<.001	12.00 (6.00-22.00)	20.99 (9.97-98.06)	<.001	0.271
ECMO/postcardiotomy shock (%)	56 (3.4)	7 (5.3)	.379	6.4 (5.1)	5.1 (5.2)	.948	0.007
Deep sternal wound Infection (%)	16 (1.0)	3 (2.3)	.342	2.1 (1.6)	2.4 (2.5)	.585	0.06
Respiratory failure (%)	193 (11.8)	65 (49.2)	<.001	28.4 (22.3)	43.9 (44.8)	<.001	0.491
Renal failure (%)	88 (5.4)	19 (14.4)	<.001	12.6 (9.9)	13.4 (13.7)	.233	0.118
Renal failure requiring dialysis (%)	20 (1.2)	9 (6.8)	<.001	3.7 (2.9)	6.2 (6.4)	.088	0.165
Pacemaker implantation (%)	70 (4.3)	9 (6.8)	.254	6.3 (5.0)	6.4 (6.5)	.478	0.067
Postoperative day of pacemaker implantation	6.00 (5.00-8.75)	14.00 (8.00-24.50)	.044	7.00 (5.00-8.71)	11.78 (4.24-21.57)	.127	0.821

Values are presented as n (%) or median (interquartile range). *ND*, Neurologic dysfunction; *SMD*, standardized mean difference; *ECMO*, extracorporeal membrane oxygenation.

**Table E15:** Multivariable logistic regression for factors associated with major postoperative complications in the aneurysm repair subgroup

Variable	Multivariable analysis		
	Odds ratio	95% CI	*P* value
Type 3 neurologic dysfunction	2.676	1.504-4.762	.001
Preoperative creatinine (mg/dL)∗	1.692	0.801-3.571	.168
Aortic insufficiency ≥ moderate (%)	0.977	0.548-1.742	.937
No cerebral perfusion	Ref	Ref	Ref
Antegrade cerebral perfusion	1.339	0.686-2.614	.392
Retrograde cerebral perfusion	0.566	0.201-1.595	.282
Antegrade + retrograde cerebral perfusion	0.845	0.307-2.331	.746
Deep hypothermic circulatory arrest time (min)	1.009	0.982-1.036	.520

*Ref*, Reference category.

∗ Values are presented as median (interquartile range).

**Table E16:** Multivariable logistic regression for factors associated with perioperative mortality in the aneurysm repair subgroup

Variable	Multivariable analysis		
	Odds ratio	95% CI	*P* value
Type 3 neurologic dysfunction	0.562	0.159-1.984	.370
Preoperative creatinine (mg/dL)∗	2.325	0.759-7.122	.139
Aortic insufficiency ≥ moderate (%)	1.460	0.437-4.875	.538
No cerebral perfusion	Ref	Ref	Ref
Antegrade cerebral perfusion	2.144	0.547-8.401	.274
Retrograde cerebral perfusion	2.728	0.396-18.794	.308
Antegrade + retrograde cerebral perfusion	1.189	0.101-14.002	.890
Deep hypothermic circulatory arrest time (min)	1.028	0.993-1.063	.115

*Ref*, Reference category.

∗ Values are presented as median (interquartile range).

**Table E17:** Multivariable Cox regression for factors associated with 11-year mortality in the aneurysm repair subgroup

Variable	Cox regression		
	Hazard ratio	95% CI	*P* value
Type 3 neurologic dysfunction	0.914	0.594-1.408	.684
Preoperative creatinine (mg/dL)∗	1.560	1.215-2.003	<.001
Major postoperative complications	2.379	1.586-3.569	<.001

∗ Values are presented as median (interquartile range).

**Table E18:** Multivariable logistic regression analyses for factors associated with NeuroARC Type 3 neurologic dysfunction in the aneurysm repair subgroup

Variable	Multivariable analysis		
	Odds ratio	95% CI	*P* value
Age	1.068	1.048-1.089	<.001
Hypertension	1.104	0.647-1.887	.716
Diabetes mellitus	1.120	0.662-1.894	.673
Dialysis	0.000	0.0-∞	.978
COPD	0.547	0.272-1.099	.090
Coronary artery disease	0.969	0.618-1.52	.891
Peripheral artery disease	1.263	0.753-2.119	.375
Congestive heart failure	1.275	0.835-1.947	.261
Preoperative atrial fibrillation	0.892	0.56-1.42	.629
Previous cardiac intervention	1.945	1.253-3.017	.003
Left ventricular ejection fraction (%)	0.981	0.963-0.998	.031
Cerebrovascular disease	2.073	1.23-3.494	.006
Isolated ascending aorta procedure	Ref	Ref	Ref
Aortic proximal extension procedure	0.771	0.372-1.599	.485
Aortic distal extension procedure	1.739	0.641-4.715	.277
Aortic proximal + distal extension procedure	1.329	0.526-3.355	.548
Concomitant CABG or mitral or tricuspid valve	0.816	0.539-1.235	.335
Cardiopulmonary bypass time	1.009	1.005-1.012	<.001
Aorta cannulation	Ref	Ref	Ref
Axillary cannulation	0.836	0.459-1.525	.560
Femoral cannulation	1.292	0.314-5.321	.723
Innominate/brachiocephalic cannulation	0.567	0.175-1.842	.345
No cerebral perfusion			
Antegrade cerebral perfusion	0.741	0.353-1.554	.428
Retrograde cerebral perfusion	3.932	1.826-8.469	<.001
Antegrade + retrograde cerebral perfusion	1.116	0.441-2.825	.816
Lowest body temperature (°C)	1.029	0.96-1.103	.416
Circulatory arrest time (min)	1.003	0.996-1.01	.452
Deep hypothermic circulatory arrest	1.117	0.49-2.545	.793

*COPD,* Chronic obstructive pulmonary disease; *CABG,* coronary artery bypass grafting; *Ref*, reference category.

**Table E19:** NeuroARC Type 3 neurologic dysfunction delirium subtype characteristics in the aneurysm repair subgroup (n = 125)

Variable	Result
Delirium etiology	
Metabolic encephalopathy	3 (2.4)
Substance withdrawal	3 (2.4)
Preoperative stroke	2 (1.6)
Other	1 (0.8)
Not documented	116 (92.8)
Delirium type	
Hypoactive	27 (21.6)
Hyperactive	74 (59.2)
Mixed	20 (16.0)
Not documented	4 (3.2)
Delirium presentation location	
ICU	106 (84.8)
SDU	14 (11.2)
Not documented	5 (4.0)
Delirium symptoms	
Agitation/restlessness	66 (52.8)
Confusion	46 (36.8)
Combative	6 (4.8)
Hallucinations/delusions	15 (12.0)
Impulsivity	2 (1.6)
Lethargic	23 (18.4)
Insomnia	10 (8.0)
Paranoia	9 (7.2)
Head CT	57 (45.6)
1	43 (34.4)
2	8 (6.4)
≥3	6 (4.8)
MRI	3 (2.4)
1	1 (0.8)
2	2 (1.6)
EEG	15 (12.0)
1	1 (0.8)
2	4 (3.2)
≥3	10 (8.0)
Neurology consultation	39 (31.2)
Psychiatry consultation	34 (27.2)

Values are presented as n (%). *ICU*, Intensive care unit; *SDU*, step-down unit; *CT*, computed tomography; *MRI*, magnetic resonance imaging; *EEG*, electroencephalogram.

**Table E20:** NeuroARC Type 3 neurologic dysfunction transient ischemic attack subtype characteristics in the aneurysm repair subgroup (n = 7)

Variable	Result
Head CT	1 (14.3)
1	2 (28.6)
2	1 (14.3)
≥3	1 (14.3)
MRI	0 (0.0)
EEG	1 (14.3)
Neurology consultation	4 (57.1)
Psychiatry consultation	1 (14.3)

Values are presented as n (%). *CT*, Computed tomography; *MRI*, magnetic resonance imaging; *EEG*, electroencephalogram.

## Appendix E1. Survival Analysis for Type 3 Neurologic Dysfunction

Overall, unadjusted 11-year survival was significantly lower in patients with Type 3 neurologic dysfunction (59.3%; 95% CI, 49.3%-71.3%) than in those without neurologic dysfunction (78.6%; 95% CI, 76.4-81.0; *P* < .001) (Figure E3).

## Appendix E2. Type 1 Neurologic Dysfunction Analysis

Unadjusted Kaplan-Meier curve showed a significant difference in 11-year survival between type 1 (T1) and no neurologic dysfunction (ND) (Figure E4). The 11-year survival of patients with T1 was 48.7% (95% CI, 38.4-61.7), which was significantly less than those without ND at 78.6% (95% CI, 76.4-81.0) (*P* < .001).

Multivariable logistic regression analysis revealed that factors independently associated with T1 included age (odds ratio [OR], 1.022; 95% CI, 1.005-1.04; *P* = .013), diabetes mellitus (OR, 1.908; 95% CI, 1.139-3.197; *P* = .014), congestive heart failure (OR, 1.701; 95% CI, 1.044-2.773; *P* = .033), vascular disease (OR, 1.693; 95% CI, 1.068-2.685; *P* = .025), aortic distal extension procedure (OR, 9.751; 95% CI, 2.036-46.709; *P* = .004), aortic proximal plus distal extension procedure (OR, 5.998; 95% CI, 1.257-28.62; *P* = .025), concomitant coronary artery bypass grafting or mitral/tricuspid valve repair (OR, 0.564; 95% CI, 0.35-0.908; *P* = .018), cardiopulmonary bypass time (OR, 1.005; 95% CI, 1.001-1.008; *P* = .005), aortic cannulation site (OR, 0.563; 95% CI, 0.334-0.949; *P* = .031), antegrade cerebral perfusion (OR, 0.246; 95% CI, 0.121-0.502; *P* < .001), antegrade and retrograde cerebral perfusion (OR, 0.330; 95% CI, 0.126-0.865; *P* = .024), and circulatory arrest (OR, 3.444; 95% CI, 1.403-8.454; *P* = .007) (Table E10).

## Appendix E3 Aneurysm Repair Subgroup Analysis

### Patient Baseline Characteristics and Operative Details

Baseline characteristics of patients who underwent aortic aneurysm repair (1770), excluding other surgical indications are shown in Table E11. In this cohort, 132 patients had Type 3 (T3) neurologic dysfunction (ND) (Figure E5). Compared with patients without ND, patients with T3 were older (70.0 vs 61.0 years; *P* < .001) and had higher rates of comorbidities, including hypertension, diabetes mellitus, coronary artery disease, peripheral artery disease, congestive heart failure, vascular disease, aortic insufficiency, cerebrovascular disease, and prior cerebrovascular accident. They also had higher median creatinine level (1.06 vs 0.98 mg/dL; *P* < .001), CHA_2_DS_2_-VASc scores (4.00 vs 2.00; *P* < .001), and more frequent prior cardiac interventions (40.9% vs 20.9%; *P* < .001) and prior myocardial infarction (11.4% vs 4.0%; *P* < .001). T3 patients underwent more urgent (28.8% vs 17.9%; *P* = .006) and emergency operations (3.8% vs 0.6%; *P* = .006).

Operative details (Table E12) showed greater use of arch procedures in the T3 group, including hemiarch (15.2% vs 10.2%; *P* < .001), partial/total arch (23.5% vs 8.7%; *P* < .001), and elephant trunk procedures (5.3% vs 1.0%; *P* = .003). T3 patients had longer cardiopulmonary bypass time (169.0 vs 136.0 minutes; *P* < .001), crossclamp time (116.0 vs 102.0 minutes; *P* = .005), and circulatory arrest time (29.0 vs 16.0 minutes; *P* < .001), lower lowest body temperatures (31.3 vs 32.0 °C; *P* < .001), and more frequent use of cerebral perfusion (50.8% vs 34.2%; *P* < .001) and deep hypothermic circulatory arrest (11.4% vs 4.7%; *P* = .002). They also had longer antegrade cerebral perfusion times (23.0 vs 13.0 minutes; *P* < .001).

### Postoperative Outcomes

Eleven-year survival for patients with T3 and no ND is shown in Figure E6. T3 patients had significantly lower unadjusted survival (60.9%; 95% CI, 49.6-74.8 vs 81.3%; 95% CI, 78.8-83.9; *P* < .001).

Propensity score weighting using overlap weights created balanced groups (127 no ND vs 98 T3) (Table E13). In-hospital outcomes are shown in Table E14. In this matched cohort, patients with T3 had higher rates of postoperative complications (including reoperation for bleeding, respiratory failure, or acute renal failure) (49.2% vs 27.6%; *P* < .001), longer ventilation times (median time, 20.99 vs 12.0 hours; *P* < .001), and higher incidence of respiratory failure (44.8% vs 22.3%; *P* < .001). T3 was found to be independently associated with major postoperative complications (odds ratio, 2.676; 95% CI, 1.504-4.762; *P* < .001) but not with perioperative mortality (odds ratio, 0.562; 95% CI, 0.159-1984; *P* = .370) (Tables E15 and E16).

Kaplan-Meier analysis showed no significant difference in adjusted 11-year survival (Figure E7)—T3 (63.2%; 95% CI, 51.7-77.2) versus no ND (62.6%; 95% CI, 57.1-68.6; *P* = .554). Predictors of 11-year survival included preoperative creatinine (hazard ratio, 1.560; *P* < .001) and major complications (hazard ratio, 2.379; *P* < .001), but not T3 (hazard ratio, 0.914; *P* = .684) (Table E17). Factors independently associated with T3 are listed in Table E18. Type 3 ND characteristics are shown in Tables E19 and E20.
